# Beyond Hydroconversion: A Paradigm Shift for Sustainable Plastic Waste Upcycling

**DOI:** 10.1021/acssuschemeng.5c04855

**Published:** 2025-06-16

**Authors:** Haokun Wang, Yiyang Li, Shik Chi Edman Tsang

**Affiliations:** The Wolfson Catalysis Centre, Department of Chemistry, 6396 University of Oxford, Oxford OX1 3QR, United Kingdom

**Keywords:** plastic upcycling, sustainability, circular economy, catalysis, hydroconversion

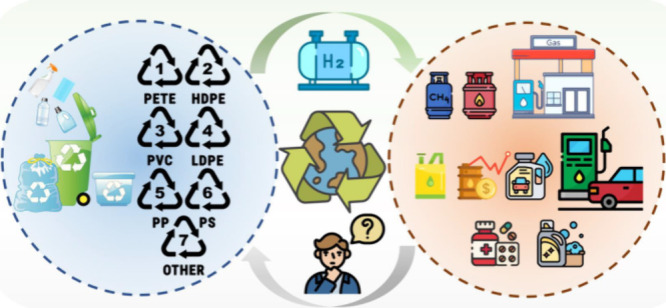

## Challenges in Current Plastic Waste Management

Plastics are indispensable to modern society, with global production exceeding 400 million metric tons in 2023.^1^ By 2050, this figure is projected to surpass 500 million metric tons, potentially consuming 10–13% of the remaining global carbon budget necessary to mitigate climate change.^2^ Despite their ubiquity, plastic waste management remains highly inefficient, with only 9% of discarded plastics being effectively recycled.^3^ Conventional disposal methods–incineration and landfilling–continue to drive environmental pollution and resource depletion.^4^

Traditional recycling methods, particularly mechanical recycling, suffer from downcycling, wherein recycled plastics exhibit inferior properties, limiting their reuse in high-performance applications.^5^ Chemical recycling techniques, such as pyrolysis and gasification, provide an alternative but require high energy inputs and often yield complex mixtures of low-value hydrocarbons, constraining their economic and industrial feasibility.^6^

Against this backdrop, catalytic plastic upcycling has emerged as a promising strategy, enabling the selective transformation of plastic waste into high-value chemicals, monomers, and hydrocarbons under relatively mild conditions. Among these, hydroconversion (e.g., hydrocracking and hydrogenolysis) has attracted significant interests due to its ability to

- Operate at moderate temperatures (∼200–300 °C), reducing energy consumption.
- Yield valuable hydrocarbons, such as naphtha, gasoline, diesel, and lubricants.
- Process a variety of plastic feedstocks with promising efficiency.

However, as plastic upcycling technologies advance, a fundamental question arises: Is hydroconversion truly the most viable long-term strategy, or does it merely perpetuate an unsustainable status quo?

## Reassessing Hydroconversion: Is It a Truly Sustainable Upcycling Strategy?

The widespread adoption of hydroconversion as a plastic upcycling strategy seems justifiable. However, does this approach genuinely align with our vision for sustainable waste management? Through extensive research in this field, we have encountered the fundamental limitations of hydroconversion that raise concerns about its long-term feasibility, particularly in terms of hydrogen dependence, energy efficiency, feedstock compatibility, and circularity.

A fundamental limitation of hydroconversion lies in its substantial reliance on molecular hydrogen, typically requiring 20–30 bar for hydrocracking and 30–60 bar for hydrogenolysis.^5^ While recent advancements in green hydrogen production–particularly photocatalytic and electrocatalytic processes–have demonstrated promise, the global hydrogen supply remains overwhelmingly dominated by fossil-derived sources, thereby entrenching dependence on nonrenewable feedstocks. As reported in 2023, despite incremental process since 2021, the global production of low-emission hydrogen remained under 1 Mtpa, accounting for less than 1% of total hydrogen output.^7^ This raises a critical question: can hydroconversion truly be considered a sustainable solution if its success hinges on nonrenewable resources? Beyond its reliance on fossil-derived hydrogen, the production of hydrogen itself is highly energy-intensive, contributing substantial CO_2_ emission unless sourced renewably. Even in scenarios where electrolytic hydrogen from renewable source is utilized, its availability and economic feasibility remain questionable at scale. Should we continue refining a process that is inherently reliant on an unsustainable input?

While hydroconversion operates at lower temperatures (<300 °C) compared to catalytic cracking (∼400 °C) and pyrolysis (∼500 °C), the energy savings it offers are incremental rather than transformative. In decentralized or low-resource settings, reliance on pressurized hydrogen and continuous energy input remains impractical. Even in industrial applications, energy efficiency improvements through hydroconversion fail to offset its fundamental dependence on fossil-based resources.

Another major challenge lies in feedstock compatibility. Hydroconversion efficiency is highly dependent on feedstock purity, with most studies reporting high efficiency only when using presorted, ultralow molecular weight plastics–a scenario that is far from the reality of heterogeneous plastic waste streams. In practical waste management, aromatic-containing additives, commonly found in commercial plastics, can irreversibly poison hydroconversion catalysts, requiring temperatures exceeding 500 °C for desorption.^8^ This raises a critical concern: Are we designing hydroconversion systems for real-world application or merely for controlled laboratory conditions?

Beyond technical feasibility, hydroconversion presents a fundamental circularity and sustainability dilemma. The primary product–fuel-range hydrocarbons such as gasoline and diesel–ultimately re-enter the combustion cycle, contributing to CO_2_ emissions. Does this genuinely advance a sustainable plastic future, or does it merely delay environmental consequences rather than eliminating them?

At what point do we move beyond incremental optimizations and pursue transformative breakthroughs that redefine plastic upcycling? As researchers, we often take comfort in small advances, yet this mindset may impede the disruptive innovations truly needed.

## Shifting the Paradigm: Hydrogen-Free Plastic Upcycling

While hydroconversion has dominated plastic upcycling research in recent years, hydrogen-free strategies offer more sustainable and energy-efficient alternatives. These approaches often enable plastic upcycling under significantly milder conditions, drastically reducing energy consumption. For instance, metathesis facilitates polymer chain scission at temperatures as low as 100 °C, providing an effective low-energy route for molecular weight reduction.^9^ Likewise, catalytic alkylation strategies have demonstrated the ability to convert polymers into gasoline-range isoalkanes under sub-100 °C conditions.^10^ Moreover, selective oxidation can proceed under comparably mild conditions (∼100–200 °C) using air or oxygen as oxidants, further minimizing energy demand.^11,12^ The use of atmospheric oxygen–an abundant, inexpensive reactant–reduces operational costs and eliminates the need for energy-intensive coreactant production. Although some tandem catalytic strategies (e.g., tandem hydrocracking/hydrogenolysis-aromatization) function at temperatures comparable to hydroconversion, their elimination of external hydrogen significantly lowers overall energy input.

From a feedstock compatibility perspective, research on the effects of additives and impurities in commercial plastics on hydrogen-free catalytic systems remains limited, but selective oxidation can proceed even in the absence of a catalyst under similar reaction conditions. This catalyst-free approach substantially mitigates the risk of catalyst deactivation from additives and containments commonly present in waste plastics. Furthermore, typical catalyst deactivation mechanisms, such as sintering and coke formation, can be mitigated or entirely avoided under air- or oxygen-rich reaction environments and at lower reaction temperatures. Consequently, these hydrogen-free strategies offer greater potential for handling complex, real-world plastic waste streams.

From both circularity and sustainability standpoints, metathesis using ethylene as a coreactant allows for the direct conversion of polyethylene (PE) into propylene, which can then be repolymerized into polypropylene (PP). This represents a far more efficient circular route compared to hydroconversion-based processes, which typically require conversion from polymer to naphtha to light olefins. Selective oxidation offers another compelling hydrogen-free upcycling strategy, transforming plastics into high-value dicarboxylic acids, which serve as key precursors for polyesters, polyols, polyamides, and nylons, as well as pharmaceuticals and industrial additives.^11–13^ In contrast to hydroconversion, which predominantly yields fuel-range hydrocarbons with limited circularity, this approach opens up a broader range of valorized products, thus enhancing both economic and industrial relevance. Furthermore, alkylation and certain tandem catalysis strategies can produce hydroconversion-equivalent products (e.g., gasoline-range products) without relying on external hydrogen. These strategies leverage thermodynamic coupling and tailored catalysts, offering significant economic and environmental advantages.

## Conclusion: The Future of Plastic Upcycling Is Hydrogen-Free Conversion

Although hydroconversion remains the dominant approach, hydrogen-free alternatives—such as metathesis, alkylation, selective oxidation, and tandem catalysis—emerge as a superior long-term solution in terms of energy efficiency, sustainability and circularity, and economic viability. Despite their promise of these methods, research efforts continue to be disproportionately focused on hydroconversion. If we are truly committed to sustainable plastic upcycling, it is essential to redirect attention toward these underexplored pathways.

The future of plastic upcycling is hydrogen-free conversion, not hydroconversion. The only obstacle to this transition is our reluctance to move beyond established paradigms.

The question is no longer whether we can use hydrogen for plastic upcycling—it is whether we should.

## Notes

'S.C.E.T. deceased on May 20, 2025.
